# How 18-month-olds with Later Autism Look at Other Children Interacting: The Timing of Gaze Allocation

**DOI:** 10.1007/s10803-023-06118-z

**Published:** 2023-08-29

**Authors:** Charlotte Viktorsson, Sven Bölte, Terje Falck-Ytter

**Affiliations:** 1 Development and Neurodiversity Lab, Department of Psychology, Uppsala University, Uppsala, Sweden; 2 Center of Neurodevelopmental Disorders (KIND), Division of Neuropsychiatry, Department of Women’s and Children’s Health, Karolinska Institutet, Stockholm, Sweden; 3 Autism Research Group (CARG), Curtin School of Allied Health, Curtin University, Perth, Australia; 4 Child and Adolescent Psychiatry, Stockholm Health Care Services, Stockholm, Sweden

**Keywords:** Autism, Eye tracking, Visual attention, Social perception

## Abstract

When observing other people during naturally paced and dynamic interactions, it is essential to look at specific locations at the right time to extract a maximum of socially informative content. In this study, we aimed to investigate the looking behavior of typically developing toddlers and toddlers later diagnosed with autism when observing other children interact. The sample consisted of 98 toddlers; 22 in a low-likelihood of autism group, 60 in an elevated likelihood of autism group who did not receive a subsequent diagnosis, and 16 in an elevated likelihood group who did receive an autism diagnosis. Participants performed an eye tracking task at 18 months of age and were assessed for diagnostic outcome at 36 months. The video stimuli consisted of two children interacting, where a boy reaches out for a toy and a girl refuses to give it to him. The low likelihood group showed an expected increase in ratio of looking at the girl’s face after the boy requested the toy, as compared to before (t(21) = -3.337, p = .003). Toddlers with later autism showed a significantly lower ratio of looking at the girl’s face during this time window, as compared to the other groups (F(2,91) = 3.698, p = .029). These findings provide new leads on how social gaze may be different in children with autism in everyday life (e.g., kindergarten), and highlight the need of studying the dynamics of gaze on short time scales.

## Introduction

In typically developing humans, social stimuli attract attention already in early infancy (Farroni, Menon, & Johnson, 2006; Gliga et al., 2009), and this preference is considered a facilitator for more complex social capacities emerging later in life (Klin, Shultz, & Jones, 2015). By looking at faces and learning from social interactions, humans gain the ability to understand what other people feel, want and believe, thus enabling social interaction and communication.

Autistic children might show challenges understanding and anticipating others’ actions. For example, in a study by Senju and colleagues (2010), 6- to 8-year-old children with autism failed to anticipate an actor’s next move in a nonverbal false belief test, where an object was placed in a box in front of an actor and then moved when the actor looked away. This pattern has also been observed in autistic adults (Schneider, Slaughter, Bayliss, & Dux, 2013). It has been hypothesized that atypical social looking behaviors in children with autism lead to a loss of information required for the development of typical social communication and interaction behaviors (for a review, see Chita-Tegmark, 2016), particularly understanding others’ actions.

**Fig. 1:**
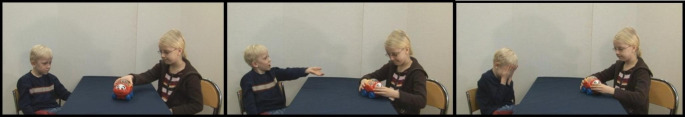
Three frames from one stimuli video, showing the girl playing with the toy, the boy reaching out for the toy, and the boy being sad after the girl refuses to give the toy

Research on social looking in autistic children has primarily focused on one-person stimuli (e.g., Sasson and Touchstone, 2014), complex stimuli consisting of multiple adults (e.g., Sasson, Turner-Brown, Holtzclaw, Lam, & Bodfish, 2008), and toddler-caregiver interactions (e.g., Shic et al., 2011). However, few studies have focused on social looking in autism in relation to viewing other children interact. This is a relevant social situation already in toddlerhood, when children are spending time in childcare settings, at playgrounds, or might be observing siblings at home. Later in development, many children are viewing other children interact throughout the day in primary school, a situation in which it is important for social learning to attend to the actions of other children. Indeed, Chevallier and colleagues (2015) found a significant difference in looking time at social stimuli between 6-17-year-old children with and without autism when viewing videos of school-aged children interacting, but not when viewing videos of non-social objects or individual faces, suggesting that further research using these kinds of stimuli is warranted. In an explorative study of the gaze behavior of typically developing children and children with autism when viewing videos of children interacting, Falck-Ytter and colleagues (2013) found a significant difference between these groups at 6 years of age. Namely, after observing a non-verbal palm-up gesture to request a toy, the typically developing children immediately focused their attention to the face of the girl holding the toy, while the children with autism showed a much weaker tendency to focus on the girl after seeing the gesture (Fig. 1). The girl holding the toy is deciding what happens next, meaning that it makes sense to look at her face to see her mimic reaction. Falck-Ytter et al. (2013) proposed that children with autism may not follow the course of such events as their typically developing peers. The same study found that more accurate performance on the eye tracking task was associated with higher non-verbal IQ in the typically developing group and associated with higher verbal IQ and communicative abilities in the autism group, suggesting that the same behavior might be linked to partially different processes in the two groups.

An important question is whether these differences in attention to other children is present already in toddlerhood. If toddlers who later receive an autism diagnosis look differently at other children interacting, they may miss social learning opportunities that could be important in preparing them for increasingly complex social interactions.

Against this background, we showed 18-month-olds with typical versus elevated likelihood of autism the same stimuli videos as in Falck-Ytter et al. (2013), while tracking their gaze. We had three pre-registered hypotheses (https://osf.io/s6epg/; *H1-H3*). It has been found that 18-month-olds are able to distinguish intentional actions of others (Behne, Carpenter, Call, & Tomasello, 2005; Olineck and Poulin-Dubois, 2005). Therefore, *H1* stated that the typically developing 18-month-olds would show similar visual patterns as the typically developing 6-year-olds in Falck-Ytter et al. (2013), namely increase their looking time at the girl’s face after the boy reaches for the toy. Secondly, *H2* stated that the toddlers who later received an autism diagnosis would show a significant difference in gaze allocation after the boy reaches for the toy, compared to the toddlers who did not receive a diagnosis. Lastly, *H3* stated that higher amount of autism traits would be associated with a lower level of looking at the girl’s face, after the boy reaches for the toy.

## Methods

### Participants

The participants were part of a prospective sibling study (Early Autism Sweden, EASE, http://www.smasyskon.se), in which infant siblings of children diagnosed with autism are followed into early childhood. The study was approved by the regional ethical review authority in Stockholm, Sweden, and was conducted in accordance with the Declaration of Helsinki.

The probability of being diagnosed with autism is increased in the case of children with an older biological sibling with the condition (Ozonoff et al., 2011); the recurrence rate is around 20%, hence the denotation ‘elevated likelihood’. Participants were recruited mainly from the greater Stockholm area. Children in the elevated-likelihood (EL) group had an older sibling or a parent with an autism diagnosis (corroborated through interview with parents and inspection of medical records), and were recruited via the project’s website, advertisement, and clinical units. Children in the low-likelihood (LL) group were recruited from birth records and through advertisements. They were typically developing, had at least one older typically developing sibling, and there were neither any parental concerns about the infant related to autism, nor any first- or second-degree family members with a history of autism. The children included in the study were reported by their parents to have no known medical conditions such as epilepsy, no known genetic syndrome associated with autism, nor any other known medical conditions affecting brain development, or visual or auditory impairment, and were born full-term (after week 36). In total, 109 participants completed both the eye tracking session at 18 months and the follow-up assessment at 36 months. Due to invalid eye tracking data, 11 participants were excluded from all analyses (see section *Eye tracking* for details). The final sample consisted of 98 children. Data included in this study were collected during the years 2014–2022.

At 18 months, all children were assessed by an experimenter, using the Mullen Scales of Early Learning (MSEL; Mullen, 1995). This developmental assessment is a standardized tool commonly used to measure early cognitive development, and it consists of the subscales gross motor ability, fine motor ability, visual reception, expressive language, and receptive language. Here we used the Early Learning Composite Score (ELCS; as a measurement of general cognitive ability), non-verbal IQ (NVIQ), and verbal IQ (VIQ). The ELCS is derived from fine motor ability, visual reception, expressive language, and receptive language. NVIQ is derived from fine motor ability and visual reception, while NVIQ is derived from the expressive and receptive language scales.

At 36 months, a comprehensive diagnostic evaluation of the children’s developmental- and adaptive abilities and autistic traits were conducted by experienced clinicians, using standardized instruments and procedures: Autism Diagnostic Observation Schedule (ADOS-2; Lord et al., 2012), Autism Diagnostic Interview-Revised (ADI-R; Rutter, LeCouteur, & Lord, 2003), Mullen Scales of Early Learning, and the Vineland Adaptive Behaviour Scale (VABS; Sparrow et al., 2005). The ADOS sessions were always either performed and coded by a child psychologist research reliable on the ADOS, or conducted by a child psychologist who later co-scored the session from video together with a research reliable colleague. The ADOS module 1 was used for 12 children, module 2 for 78 children, and module 3 for one child, and therefore the standardized comparison score was included in all analyses. Categorical diagnostic assessments were based on DSM-5 (American Psychiatric Association, 2013). After this assessment, participants were divided into three groups: (1) EL-ASD, elevated likelihood and autism diagnosis, (2) EL-noASD, elevated likelihood but no autism diagnosis, and (3) LL, low likelihood. No children in the LL group were diagnosed with autism at the 36-month assessment. Our final sample consisted of 16 children in the EL-ASD group, 60 children in the EL-noASD group, and 22 children in the LL group. See Table 1 for descriptive statistics. Ethnicity of the parents, but not of the children, was collected. The reported ethnicity of mothers (n = 92) were: White (95.7%), Asian (2.2%) and Other (2.2%). The reported ethnicity of fathers (n = 90) were: White (92.2%), Mixed (4.4%), Other (2.2%), and Asian (1.1%). It is notable that approximately 27% of the population in Stockholm was born outside of Sweden (www.scb.se), suggesting that our sample might not reflect the diversity of the area.

**Table 1:** Descriptive statistics

		Mean (SD)a			
		[Min – Max]			
	Total (n = 98)^b^	LL (n = 22)	EL-noASD (n = 60)	EL-ASD (n = 16)	P^c^
N females (%)	45 (45.9%)	11 (50.0%)	29 (48.3%)	5 (31.3%)	-
Age (in days)	559.4 (19.7)	562.9 (25.3)	557.9 (17.3)	560.1 (20.0)	0.586
	[506–643]	[520–643]	[506–599]	[532–617]	
Parental education^d^	3.25 (.71)	3.28 (.70)	3.30 (.74)	3.06 (.60)	0.507
	[1.5–4.0]	[2.0–4.0]	[1.5–4.0]	[2.0–4.0]	
MSEL (18 months)^e^					
ELCS	95.93 (13.80)	99.67 (13.65)	97.64 (12.85)	84.47 (12.14)	0.001
	[65–123]	[69–119]	[76–123]	[65–106]	
NVIQ	106.33 (10.24)	108.05 (11.16)	107.04 (9.32)	101.27 (11.35)	0.104
	[83–128]	[85–128]	[83–128]	[83–122]	
VIQ	94.02 (18.92)	100.48 (17.40)	96.16 (17.71)	77.13 (16.52)	< .001
	[40–138]	[71–138]	[67–132]	[40–105]	
ADOS (36 months)					
Total score	3.69 (2.22)	2.45 (1.44)	3.45 (1.89)	6.85 (1.73)	
	[1–10]	[1–6]	[1–8]	[4–10]	
RRB score	5.70 (2.48)	4.64 (2.15)	5.39 (2.24)	8.85 (1.41)	
	[1–10]	[1–7]	[1–9]	[6–10]	
SA score	3.80 (2.11)	2.86 (1.81)	3.68 (2.04)	5.92 (1.44)	
	[1–8]	[1–7]	[1–8]	[4–8]	

^a^ Except for N females, which shows the frequency

^b^ n for individual variables varies slightly (90–98)

^c^ One-way analysis of variance F-tests to test group differences, using significance level 0.05

^d^ Education level on a scale from 1 to 4, where 1 = Primary, 2 = Secondary, 3 = Undergraduate (≤ 3 years) and 4 = Postgraduate level (> 3 years), averaged for both parents

^e^ MSEL: Mullen Scales of Early Learning, ELCS: Early Learning Composite Score, NVIQ: Non-verbal IQ, VIQ: Verbal IQ.

### Experimental Stimuli

The participants were shown eight videos (12–18 seconds long) in a pseudo-random order, in which two young children were engaged in toy directed activities and interacted using non-verbal gestures (Fig. 1). Half of the videos were duplicates, where the original video was flipped (i.e., the girl sitting to the left instead of to the right). In each video, the girl plays with a toy. The boy then asks for the toy using non-verbal gestures, but the girl refuses to give it. The boy then shows clear disappointment and pretends to cry. In half of the videos the girl ignores the boy after she refuses to give the toy (here named the no tease condition, but called no give condition in Falck-Ytter et al., 2013), and in half of the videos she teases him using non-verbal gestures and facial expressions (tease condition). The videos were accompanied by natural sounds, such as vocalizing and crying, but the children did not speak. In half of the videos the toy consisted of a car and in half of the videos it consisted of a dinosaur. These videos were selected based on the results by Falck-Ytter et al. (2013), who found that the gaze behavior of preschool age autistic children differed significantly from the gaze behavior of typically developing children when viewing these stimuli videos. Because all children in the EASE study have at least one older sibling, and therefore probably are used to seeing older children playing, we deemed the stimuli to be useful in this sample as well. We included both the tease and the no tease condition, since children already at ages 8–18 months have been found to understand different forms of teasing (Colle, Grosse, Behne, & Tomasello, 2023; Reddy and Mireault, 2015).

### Eye Tracking

The eye-tracking task in the current study was only included at the 18-month visit. Here, the families visited the lab for a full day with several assessments and experiments. During the eye-tracking session (which lasted about 10 minutes and also included stimuli linked to other experiments) the child was placed in their parent’s lap at approximately 60 cm from the screen. A five-point calibration was conducted before eye-tracking recording, by directing the toddlers’ attention to a series of positions on the screen using moving stimuli. The calibration procedure was repeated if necessary and the experiment did not start until a successful calibration was made. Gaze data was collected using eye-trackers Tobii 1750 and Tobii TX300.

**Fig. 2:**
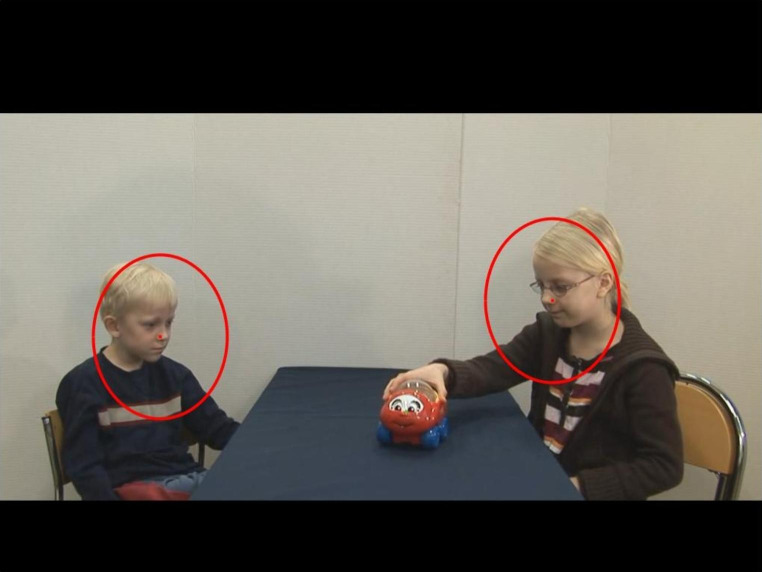
Areas of interest (AOIs) plotted on a frame from one of the stimuli videos. The center of each AOI is marked with a dot. Each AOI is 100 × 80 pixels

Data were analyzed using custom scripts written in MATLAB (available upon request). Areas of interest (AOIs) are shown in Fig. 2. They were dynamic and coded manually for each frame. The ratio of looking at the face of the girl after the boy reaches for the toy was calculated as looking time at the girl AOI during a 3-second time window after the boy has extended his arm, divided by looking time at the whole screen during the same time period. The same procedure was used for calculating the ratio of looking at the face of the girl during a 3-second time period before the boy reaches for the toy. Average looking time at the screen was also calculated, as well as mean ratio of looking at both the girl and the boy, during the whole videos (as a measure of preference for looking at faces in general).

Each time the boy requested the toy using a palm-up gesture was classified as one trial. There were two instances of the boy reaching for the toy in each video, meaning that there were 16 trials in total. A trial was classified as invalid if the child looked less than 1000 milliseconds on the screen in the 3-second time window *before* the boy reaches for the toy, or if they looked less than 1000 milliseconds on the screen in the 3-second time window *after* he reached for the toy. Trials were also classified as invalid if the child looked at the screen for less than 50% of the length of the whole video. If both trials in a video were invalid, all data from that video (such as mean looking time at screen) were excluded from further analyses. If a participant had more than 8 invalid trials, the participant was excluded from all further analyses. Following these criteria, eleven children were excluded (two from the LL group, three from the EL-ASD group, and six from the EL-noASD group). There were no statistically significant differences between the excluded and included participants regarding age (t(107) = 1.179, p = .241), sex (t(12.735) = -1.314, p = .212), or parental education (t(106) = 0.000, p = 1.00).

### Primary Statistical Analyses Linked to Hypotheses H1-3

Paired-samples t-test were used to test whether the LL group differed in total looking time and ratio of looking at the face of the girl before and after the boy reached for the toy. Group differences were tested using a one-way ANOVA if the assumption of equal variances was met, otherwise a Welch test was used. Sex, age, parental education (as a proxy for socio-economic status), and number of excluded trials were included as covariates. Eight participants did not provide data for parental education, in these cases the total sample mean (3.25) was used. Statistical tests linked to preregistered hypotheses (H1 – H3 and planned comparisons) were not corrected for multiple testing. Bonferroni correction was used for exploratory post-hoc group comparisons. Associations with autism traits were calculated using Pearson’s r.

### Exploratory Analyses Using the D2R Measure

**Fig. 3:**
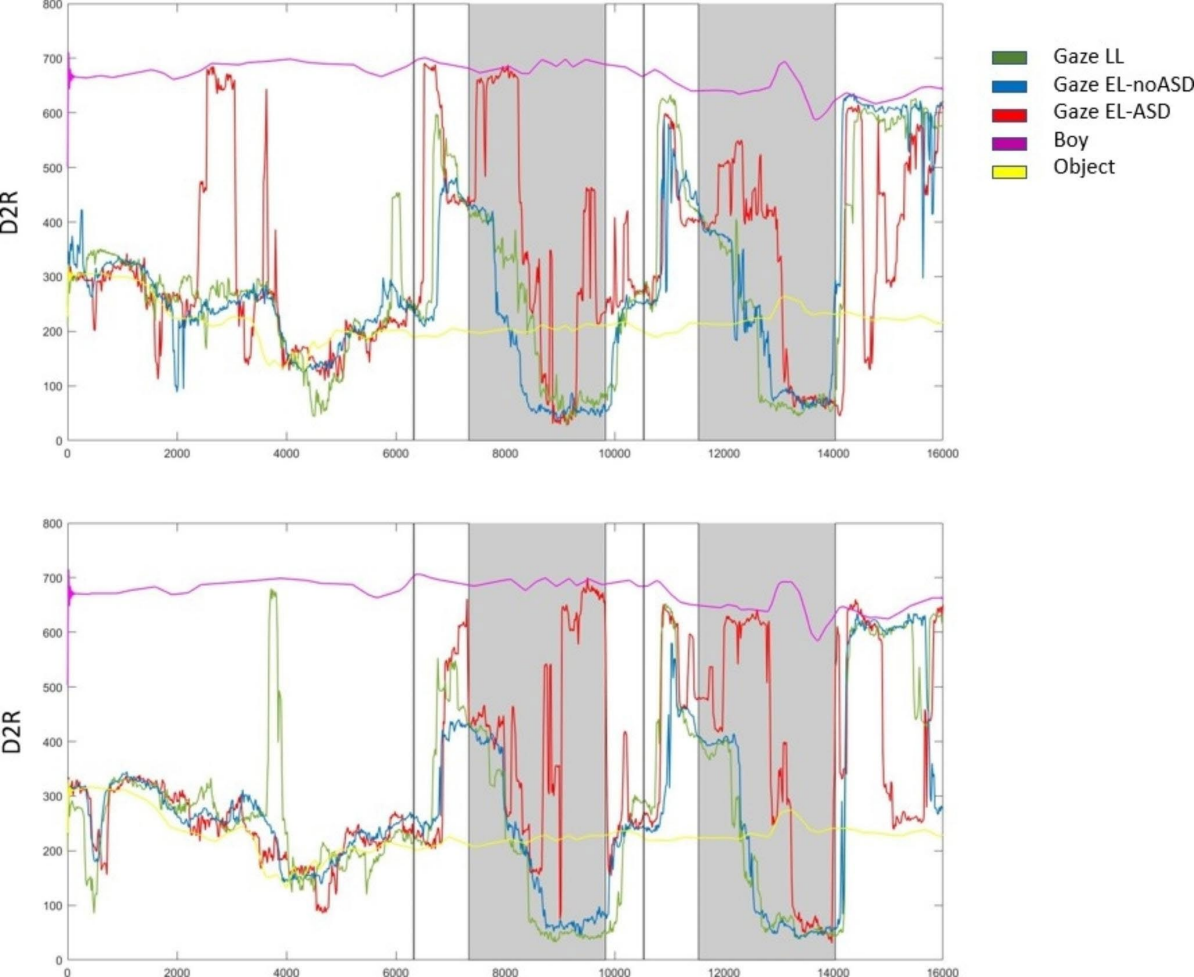
A visualization of gaze data for LL (green), EL-noASD (blue), and EL-ASD (red) during two stimuli videos. The x-axis represents time (milliseconds) and the y axis represents the median Euclidian distance (i.e., distance to reference point, D2R, in pixels) to the girl’s face for each group. The distances from the toy (yellow) and the boy’s face (purple) to the face of the girl are also shown. The vertical black lines mark the boy’s reach for the toy and the shaded areas are the delayed time window used in the follow-up analyses

In line with the procedure in Falck-Ytter et al. (2013), we wanted to explore other potential time frames in the videos where the gaze behavior of the three groups differ (in relation to the girl’s face but unrelated to AOIs). Due to the low number of studies analyzing the temporal timing of gaze allocation at this age, there might be other specific points in the videos where the groups differ in their gaze preference, in ways we might not have anticipated. Therefore, we also measured the Euclidian distance from each individual’s gaze point to the center of the girl’s face for each frame in each video. By using the distance to reference point (D2R; Falck-Ytter et al., 2013) statistic, it is possible to visualize complex temporospatial relations in a simple two-dimensional plot (see Fig. 3). Here, the girl’s face is the reference point, and the two-dimensional plot shows the distance to her face throughout the videos. We then calculated the median D2R for each individual during 1-second time bins throughout all videos, in line with the methodology in Falck-Ytter et al. (2013). By using 1-second time bins, potential group differences can be studied throughout each video while keeping the number of analyses down and increasing the chance of having valid data from all participants (as compared to when using shorter time windows). Group differences for each time bin were calculated using one-way ANOVA’s. Bonferroni correction was used to correct for multiple testing. The total number of tests was 126, meaning that the corrected p value was 4.0 × 10^− 4^.

## Results

### Hypothesis H1 and H2

There were no statistically significant group differences in number of excluded trials (F(2,95) = 0.035, p = .966) or mean looking time at screen (F(2,33.4) = 1.272, p = .294). In the LL group, the mean ratio of looking at the girls’ face was higher before the reach (0.595, SD = 0.102) than after the reach (0.518, SD = 0.120), and this difference was statistically significant (t(21) = 4.837, p < .001), indicating that, against *H1*, this group decreased their looking at the girl’s face after seeing the request from the boy in the pre-specified time interval.

Given the surprising finding in the prespecified analysis of the LL group (*H1*), we decided to visualize the gaze behavior in the three groups using the D2R metric, in order to discern whether group differences might occur at other time intervals. As can be seen in Fig. 3, the children in fact exhibited the expected pattern of increased looking time at the girl after the boy reached for the toy, but this happened somewhat later than our pre-specified time interval. This was presumably due to the young age of the children, compared to the previous study of 6-year-olds (Falck-Ytter et al., 2013). We therefore decided to analyze the gaze data within a 2.5 s time window starting one second *after* the reach (the shaded area in Fig. 3), and comparing it to a 2.5 s time window immediately before this (i.e., 1.5 s before the reach plus one second after the reach). A shorter time range of 2.5 s was used in order to avoid including the next request for the toy. In other words, we followed the steps in our preregistered plan, but with a slightly delayed time window to adjust for the putative effect of the young age of the toddlers.

**Fig. 4:**
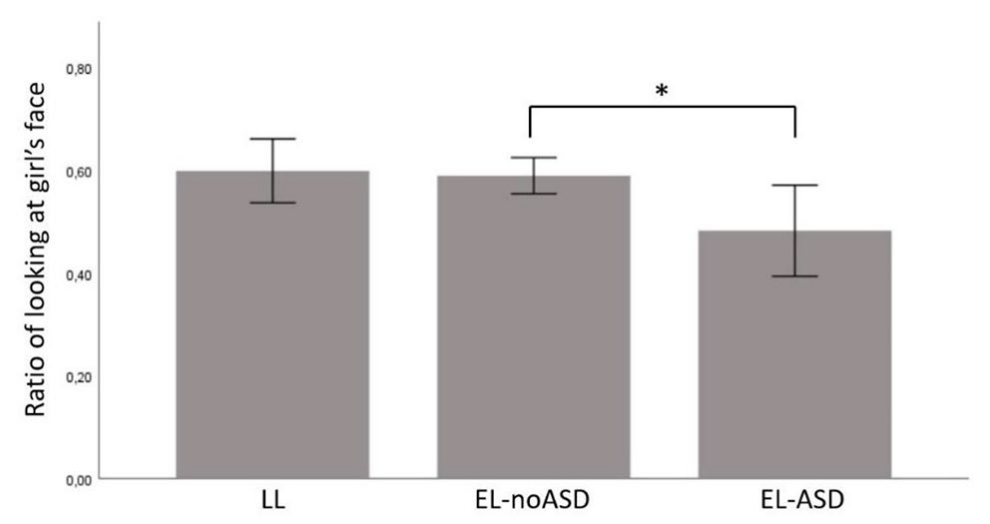
Group means for ratio of looking at the girl’s face after the boy requests the toy; * = p < .05

Using this adjusted time window, the LL-group showed the expected increase in the ratio of looking at the face of the girl before (mean = 0.530, SD = 0.095) versus after (mean = 0.599, SD = 0.141) the onset of the delayed time window (t(21) = -3.337, p = .003). See Table 2 for descriptive statistics of eye tracking measures. When testing *H2*, we found a statistically significant group difference during the delayed time window (F(2,91) = 3.698, p = .029; see Table 3) regarding ratio of looking at the girl’s face, but no statistically significant difference between the groups before the delayed time window (F(2,91) = 0.054, p = .947). Planned comparisons showed a statistically significant difference between the EL-ASD group (mean = 0.482, SD = 0.166) and the EL-noASD group (mean = 0.589, SD = 136; p = .037), but not between the EL-ASD group and the LL group (mean = 0.599, SD = 0.141; p = .053), but it is notable that, descriptively, the mean difference between these groups were slightly larger (see Fig. 4).

**Table 2:** Descriptive statistics of eye tracking measures

		Mean (SD) [Min – Max]		
	Total (n = 98)	LL (n = 22)	EL-noASD (n = 60)	EL-ASD (n = 16)
Number of excluded trials (0–8)	1.14 (2.05) [0–8]	1.23 (2.20) [0–8]	1.10 (2.09) [0–7]	1.19 (1.80) [0–6]
Mean looking time at screen (seconds)	13.35 (1.65) [8.43–15.16]	13.59 (1.37) [10.63–14.92]	13.44 (1.62) [8.43–15.16]	12.66 (2.03) [9.09–14.93]
Mean ratio of looking at girl (after reach)	0.574 (0.146) [0.168 – 0.841]	0.599 (0.141) [0.208 – 0.822]	0.589 (0.136) [0.168 – 0.841]	0.482 (0.166) [0.232 – 0.747]
Mean ratio of looking at girl (before reach)	0.532 (0.106) [0.304 – 0.794]	0.530 (0.095) [0.304 – 0.694]	0.536 (0.109) [0.325 – 0.749]	0.518 (0.113) [0.315 – 0.794]
Mean ratio of looking at both faces	0.787 (0.056) [0.665 – 0.902]	0.802 (0.060) [0.704 – 0.894]	0.785 (0.054) [0.665 – 0.896]	0.782 (0.059) [0.706 – 0.902]

**Table 3:** F-values and p-values for the ANOVA’s of the effect of group (including covariates) on the ratio of looking at the girl’s face before and after the request for the toy

	Before reach		After reach			
	F	p	F	p		
Group	0.054	0.947	3.698	0.029*		
Age	9.654	0.003*	0.265	0.608		
Sex	5.734	0.019*	2.328	0.131		
Parental education	0.009	0.923	0.253	0.616		
Number of excluded trials	1.034	0.312	0.016	0.898		

*p < .05

### Follow-up Analyses Linked to Hypothesis H1 and H2

Due to the high similarity between all videos, we explored potential learning effects by comparing the first (valid) trial and the last (valid) trial for each participant. These analyses were not pre-registered and the corrected significance value was 0.017 (three scales). A paired-samples t-test showed no statistically significant difference in the ratio of looking at the girl’s face during the delayed time window for the LL group (t(21) = − 0.271, p = .789), the EL-noASD group (t(59) = − 0.899, p = .372), or the ASD group (t(15) = − 0.309, p = .762), suggesting that performance was similar for the early and late trials.

In order to explore whether the group difference in the ratio of looking at the girl’s face during the delayed time window changed over toy condition (car vs. dinosaur) and tease condition (no tease vs. tease condition), we performed two mixed ANOVAs. These analyses were not pre-registered, and the corrected alpha value was 0.025 (two scales).There were no statistically significant interaction effects between group and toy (F(2) = 2.489, p = .089) or group and tease/no tease (F(2) = 0.171, p = .844), suggesting that the group difference was present regardless of which toy was used, and regardless of whether the girl ignored the boy or teased him after he reached out for the toy (see Supplementary Information Figure S1). There was no main effect of condition (F(2) = 1.584, p = .211) or toy (F(2) = 0.267, p = .607).

In order to explore whether there might be a group difference in general preference for looking at faces during the total extent of the videos, we tested group differences for the mean ratio of looking at both the girl and the boy (relative to the whole screen). There were no statistically significant group differences in this measure (F(2,91) = 0.649, p = .525).

In addition, in an analysis prompted during the peer review process (and hence not pre-registered), we analyzed the latency of looking towards the face of the girl after the boy reaches for the toy, as well as the duration of the first fixation at her face after the reach. The corrected alpha value was 0.025 (two scales). No statistically significant difference between groups was found for mean latency (F(2,91) = 2.148, p = .123) or fixation duration (F(2,91) = 0.181, p = .835), see Supplementary Information S1 for additional information.

### Hypothesis H3

For both the whole sample and the total combined elevated likelihood group, the association between the ADOS-2 total comparison score and the ratio of looking at the girl’s face during the delayed time window was negative but non-significant (r = − .176, p = .095 and r = − .125, p = .307, respectively). In order to explore whether there might be a specific association with a subscale of ADOS-2, we calculated the correlation between ratio of looking at the girl’s face and the SA (social affect) and the RRB (repetitive and restrictive behavior) subscale comparison scores focusing on the combined elevated likelihood group. These analyses were not pre-registered, and the corrected alpha value was 0.025 (two scales). The RRB subscale showed a statistically significant negative association with ratio of looking at the girl’s face (r = -,0.285, p = .018), but the SA subscale did not (r = − .054, p = .660).

In addition, we calculated the association between the ratio of looking at the girl’s face during the delayed time window and non-verbal and verbal IQ, separately for each group. These analyses were not pre-registered, and the corrected alpha value was 0.008 (six scales). Non-verbal IQ showed a positive association with ratio of looking at the girl’s face in the LL group (r = .478, p = .028), which was not statistically significant after correction for multiple testing. There was no statistically significant association between non-verbal IQ and ratio of looking at the girl’s face in the EL-noASD group (r = − .160, p = .238) or the EL-ASD group (r = .059, p = .836). Verbal IQ was not associated with ratio of looking at the girl’s face in the LL group (r = .402, p = .071), the EL-noASD group (r = − .027, p = .847), or the EL-ASD group (r = .105, p = .711).

### Exploratory Analyses Using the D2R Measure

After correcting for multiple testing, only two 1-second time bins showed statistically significant differences between groups in the median distance to the girl’s face (see Supplementary Information Table S1). Both of these time bins occurred within the delayed time window after the boy reached for the toy. No other pattern of differences among videos was detected.

## Discussion

This study suggests that when observing other children interacting, at certain moments in time toddlers later diagnosed with autism may look less than neurotypical children at locations that are maximally indicative of what is going to happen next. Specifically, 18-month-old children with later diagnosed autism showed a significantly lower ratio of looking at the face of a girl holding a toy, after a boy non-verbally requested it, compared to the children who were not subsequently diagnosed with autism. This was true even when controlling for age, gender, number of included trials, and parental education. There was no difference in the ratio of looking at the girl in the first and the last trial for any of the groups, indicating that this looking behavior was not an effect of learning. The difference between groups was not moderated by the type of toy used, or whether the girl teased the boy or not, suggesting that the difference between the children is not due to low-level visual cues, such as facial expression or the appearance of the toy. There were no group differences in the ratio of looking at both the girl and the boy for the duration of the whole videos, indicating that there is not a generally lower motivation to look at faces in the autism group. Rather, it suggests that toddlers who later develop autism look less at the most informative aspect of the scene, i.e. the face of the girl, at a particular moment in time. Looking at her after the boy’s request is likely to be adaptive from an information-seeking point of view, because she holds information on why she did not hand over the toy and what she is going to do next. Indeed, that is the pattern displayed by the children in the low-likelihood group, who significantly increased the ratio of looking at the girl’s face after the boy reached for the toy. If toddlers who later develop autism look differently at other children interacting, they may miss learning opportunities that could prepare them for increasingly complex social interactions. This might lead to difficulties in anticipating other’s actions (Schneider et al., 2013; Senju et al., 2010), and could ultimately have consequences for their ability to interact with other people (von Hofsten, Uhlig, Adell, & Kochukhova, 2009). Notably, disagreements and small conflicts (such as the one displayed in our stimuli) are common among children, and learning from these situations is an important aspect of social development. Although a statistically significant difference was found only between the children with elevated likelihood of autism that did and did not get an autism diagnosis later in life, it is notable that the mean difference was even higher between the low likelihood group and the autism group. It is therefore likely that this non-significant finding is a result of low power, due to small sample sizes in these groups.

What could be the reason behind the observed group difference in looking towards the girl’s face just after having seen the boy’s gesture? Difficulties understanding and anticipating other’s actions have been found in both children and adults with autism (Senju et al., 2010; Schneider et al., 2013), while typically developing children have been found to understand others’ intentions already at 18 months of age (Behne, Carpenter, Call, & Tomasello, 2005; Olineck and Poulin-Dubois, 2005). Therefore, it is possible that the group difference we found in this study is due to an underlying difference in the ability to understand the intentions of the children playing with the toy. Another possibility is that autistic toddlers are slower in disengaging gaze from fixated stimuli in general (Elsabbagh et al., 2013; Zwaigenbaum et al., 2005). However, it would imply a general pattern of slow disengagement in the autism group throughout the duration of the videos, which is not consistent with the fact that the difference between groups were only found at very specific instances. Furthermore, we did not find a statistically significant group difference in the duration of the first fixation at the girl’s face after the boy reached for the toy, suggesting the same level of disengagement in all groups. Another possibility is that toddlers later diagnosed with autism have difficulties understanding the gesture the boy makes when he requests the toy. Although earlier research has found that 14-month-olds with typical development recognize the communicative gesture of a palm-up request between two other people, and that they can anticipate how a third-party addressee will respond to the gesture (Thorgrimsson, Fawcett, & Liszkowski, 2014), it has been found that infants with later autism use fewer gestures than typically developing infants (Choi, Shah, Rowe, Nelson, & Tager-Flusberg, 2020; Watson et al., 2013), and that they show less variety in type of gestures used (Colgan et al., 2006). Although speculative, it is possible that difficulties understanding the gesture or the situation more generally is linked to general atypicalities in predictive abilities (Sinha et al., 2014). According to some of these accounts, autistic individuals may have difficulties with prediction in certain environments (Lawson, Rees, & Friston, 2014; Van de Cruys et al., 2014). The complexity and unpredictability of social interactions may make such situations most noticeably affected by the difference in information processing. This might explain the findings in this study, but more studies are needed in order to discern the ability of toddlers with later autism to predict and understand others’ actions based on social gestures and non-verbal communication. It is notable that children with autism do not generally experience challenges predicting physical events or the goal of simple actions with their gaze (von Hofsten et al., 2009; Achermann et al., 2021; Falck-Ytter, 2010; see also Falck-Ytter et al., 2023). Notably, the association between the ratio of looking at the girl’s face after the gesture (measured at 18 months) and the RRB subscale of the ADOS-2 (measured at 36 months) indicates that the tendency to look at her face reflects differences in non-social traits, such as predictive abilities, restrictive interests or lower level sensory differences. It is possible, for example, that children scoring high on RRB have variable looking styles reflecting their individual and generally stronger sensory preferences or interests, which at most times do not surface as group differences due to heterogeneity in looking also among other children. However, after the boy’s gesture, homogeneity increases in non-autistic children’s looking behavior. At this moment in time, looking in children with high RRB scores may remain more scattered not because they do not understand the gesture or have lower social motivation, but because they have a higher interest in other events and objects.

In this study we found a similar gaze pattern in the low likelihood group at 18 months of age as Falck-Ytter et al. (2013) found in 6-year-old typically developing children, suggesting that the response to orient towards the face of the girl in this situation is present already in toddlerhood, indicating an early understanding of social gestures and non-verbal communication (Thorgrimsson et al., 2014). Falck-Ytter and colleagues (2013) found that higher non-verbal IQ predicted more looking towards the face of the girl after the boy requested the toy, but only in the typically developing group. Similarly, we found a positive association between non-verbal IQ and ratio of looking at the girl’s face after the boy reached for the toy in the low-likelihood group, but this association was not statistically significant. Falck-Ytter et al. (2013) found that looking time at the girl’s face was predicted by verbal IQ in the autism group, but we did not observe an analogue association in the current study. This might be due to the age of the participants, since they have very recently started developing language at 18 months of age.

We did not find a statistically significant association between the ratio of looking at the girl’s face after the palm-up gesture at 18 months and ADOS-2 total comparison scores at 36 months for either the total sample or the elevated likelihood group (including both children that did and did not develop autism). In the elevated likelihood group, there was no association between ratio of looking at the girl’s face and the SA (social affect) subscale, but we found that higher ratio of looking at the girl’s face (after the reach) was associated with less restrictive and repetitive behaviors (RRB subscale). Notably, this might suggest that the difference in gaze behavior between the autism group and the other toddlers when observing this social scene is in fact not related specifically to difficulties with social communication, as measured by the ADOS-2. Therefore, it might not be plausible that the observed group difference is due to the ability to understand the intention of others, or to decode the gestures used in the videos.

In infant and child eye tracking research, it is common to use measures of gaze behaviors that are based on aggregated looking time. However, this study highlights the importance of studying gaze behavior during shorter time windows, to find specific instances of differences in gaze allocation and timing of gaze shifts. Here, we found a clear pattern of differences after a specific event in the video, rather than finding a general tendency to orient less towards faces in the autism group. An important goal of future studies of autism should therefore be to look at specific instances of divergent gaze behavior in social and non-social situations, instead of merely analyzing aggregated looking time.

One limitation in the current study is the high similarity among stimuli videos. Although this is useful for studying the stability of a specific gaze behavior (which was the primary interest of this study), it limits the possibility to explore potential group differences when viewing other forms of social scenes. A priority for future studies should be to study the timing of gaze allocation to important aspects of a diverse set of social scenes, in both children with and without later autism. For example, different forms of gestures and interactions should be incorporated. This might elucidate the specificity and extent of this behavior, leading to a deeper understanding of how children with later autism look at social scenes and how that might affect later development. Another important limitation is the small sample size, leading to a potential lack of power to detect differences between groups. Although longitudinal infant sibling studies are time-consuming and costly, it is vital that we collect as much data as possible. Future studies of social looking in toddlers with later autism should aim for larger sample sizes and data collection at more time points.

## Conclusions

This study suggests that when observing other children, toddlers subsequently diagnosed with autism sometimes look less at the most socially informative locations, but that moment-to-moment analysis of gaze is necessary to understand under which specific circumstances this occurs. Typically developing toddlers increase the ratio of looking at a girl’s face after a boy non-verbally requests a toy she holds, while toddlers with later autism do not show this gaze pattern. This difference in temporal timing of gaze allocation might lead to altered information processing and may have cascading consequences for development and for learning from social observation and interaction.

## Data Availability

Data, code, and materials are not publicly accessible, but will be made available upon reasonable request to corresponding author. Note that sharing of pseudonymized personal data will require a data sharing agreement, according to Swedish and EU law. The analyses were preregistered (https://osf.io/s6epg/).
